# Molecular Detection of Feline Coronavirus Based on Recombinase Polymerase Amplification Assay

**DOI:** 10.3390/pathogens10101237

**Published:** 2021-09-25

**Authors:** Rea Maja Kobialka, Arianna Ceruti, Michelle Bergmann, Katrin Hartmann, Uwe Truyen, Ahmed Abd El Wahed

**Affiliations:** 1 Institute of Animal Hygiene and Veterinary Public Health, Leipzig University, 04103 Leipzig, Germany; rea_maja.kobialka@uni-leipzig.de (R.M.K.); arianna.ceruti@uni-leipzig.de (A.C.); truyen@vetmed.uni-leipzig.de (U.T.); 2 Clinic of Small Animal Medicine, LMU, 80539 Munich, Germany; N.Bergmann@medizinische-kleintierklinik.de (M.B.); hartmann@medizinische-kleintierklinik.de (K.H.)

**Keywords:** recombinase polymerase amplification, diagnostic, feline coronavirus, FIP, feline infectious peritonitis, RT-RPA, point-of-need testing

## Abstract

Feline coronavirus (FCoV) is endemic in cat populations worldwide. Persistently, subclinically infected cats play a significant role in spreading the infection. Testing fecal samples of cats may facilitate efforts to decrease the viral burden within a population. Real-time RT-PCR is highly sensitive and specific for the detection of FCoV but must be performed in a fully equipped laboratory. A simple and accurate assay is needed to identify FCoV at the point-of-need. The aim of this study was to develop a rapid FCoV detection assay based on isothermal amplification technology, i.e., reverse transcription-recombinase polymerase amplification (RT-RPA). Primers were designed to target the highly conserved 3′ untranslated region of the 7b gene. Running on a constant temperature of 42 °C, reverse transcription as well as DNA amplification and detection was achieved in a maximum of 15 min. A probit analysis revealed a detection limit of 58.5 RNA copies/reaction. For cross-detection, nucleic acids from 19 viruses were tested. Both RT-RPA and real-time RT-PCR showed cross-detection with canine coronavirus and transmissible gastroenteritis virus, but not with other pathogens. To evaluate clinical performance, RNA was extracted from 39 fecal samples from cats. All samples were tested simultaneously with real-time RT-PCR resulting in a RT-RPA sensitivity and specificity of 90.9% and 100%, respectively. RT-RPA can be considered a promising simple method for rapid detection of FCoV.

## 1. Introduction

Feline coronavirus (FCoV) is an enveloped single-stranded positive-sense RNA virus, belonging to the genus *Alphacoronavirus*, family *Coronaviridae*, and order *Nidovirales* [1]. Based on pathogenicity and gene mutations, FCoV can be divided into two biotypes: the common avirulent feline enteric coronavirus (FECV) and the highly virulent feline infectious peritonitis virus (FIPV) [2,3]. The clinical signs of feline infectious peritonitis (FIP) were first described in cats in the US in 1963 [4]. Since then, FCoV has been reported worldwide and not a single cattery can be considered free from the virus [5]. FCoVs are subcategorized based on reactivities of neutralizing antibodies to two serotypes, I and II [6]. Serotype I is more prevalent than serotype II in most cat populations tested [7,8,9,10]. Natural transmission of FECV is predominantly via the fecal-oral route, with subsequent infection of epithelial cells, mostly enterocytes [1,11]. It is known that multi-cat environments show a higher prevalence of FCoV infections than single-cat environments and different factors are discussed to play an important role in the spread of the virus [12,13,14]. One suspected cause is the frequent use of shared litter trays in facilities with several cats. If one cat is infected, FCoV spreads very quickly within this population [15,16,17]. In addition, subclinical shedders are a big problem, as they often remain undetected and represent a possible source of infection [16,18]. Furthermore, the age of the cats is important, as kittens under one year of age are known to shed FCoV in high amounts [5,14,17,19]. Generally, most FCoV-infected cats remain without clinical signs or only develop mild enteric signs. Sudden changes in the virus tropism to macrophages and monocytes instead of enterocytes due to mutations in the spike gene [20] lead or contribute to the development of the fatal systemic disease, FIP, in 5–12% of infected cats in multi-cat environments [21]. The clinical signs of FIP are mostly non-specific, including apathy, fever and weight loss, often followed by cavity effusions and/or neurological or ocular signs [15]. Cats with effusive FIP only have a median survival time of a few days [22]. New studies with antiviral therapy resulted in promising results, but the medication is only available for routine clinical use in few countries so far [23,24].

Several molecular assays based on RT-PCR, either conventional or real-time, were established for the detection of FCoV [25,26,27,28]. Target samples are usually thoracic or abdominal effusion, blood and/or feces. It is important to emphasize that the detection of FCoV in the latter is indicative of FECV infection and has little diagnostic value for the diagnosis of FIP infection [29]. RT-PCR is highly accurate in detecting FCoV in the feces of cats with and without clinical signs [16]. However, to maximize chances of detecting intermittent shedders, it is recommended to perform RT-PCR on at least three fecal samples collected at intervals between one week and one month [5]. One drawback of PCR assays is that they must be performed in centralized, highly equipped laboratories by trained technicians. A simple, easy to use rapid point-of-need test would be useful to identify and isolate subclinically infected FCoV shedders to decrease the disease burden. Promising molecular-based methods are isothermal amplification technologies, e.g., the loop-mediated isothermal amplification (LAMP) [30,31] or recombinase polymerase amplification (RPA) [32]. RPA has been used to detect the nucleic acid sequences from a wide variety of pathogens within a 15 min assay run-time [33,34,35,36,37]. The rapid results were achieved by using additional proteins to separate the DNA strands instead of thermal cycling as in the PCR. The simplicity of the RPA assay enables the use of a portable detector device [38].

In this study, the RT-RPA assay for rapid FCoV detection was developed based on the highly conserved 3′UTR of the 7b gene. The sensitivity and specificity to detect FCoV as well as cross-detection was determined. The assay’s clinical performance was validated using fecal samples. All results were compared with real-time PCR as a reference method.

## 2. Results

### 2.1. Selection of RPA Primers and Probe

To select the most efficient primer combination that could amplify few copies of FCoV RNA, nine combinations of RPA primers were screened using a concentration of 10^5^ and 10^2^ of the RNA molecular standard/μL (Figure S1). To identify any non-specific fluorescence signals, a negative control containing only molecular grade water as a template was used (Figure S2). The threshold time (TT) in seconds was calculated as the first rise of fluorescence intensity Millivolt (mV) above the baseline (the fluorescence intensity in the first minute) in the first derivative analysis. The best TT value of 180 and 300 s and a fluorescence signal of 4327 mV and 3481 mV for the 10^5^ and 10^2^ RNA/reaction, respectively, were achieved using Forward Primer 1 (FP1) and Reverse Primer 3 (RP3) (Figure S1). FP1 + RP3 primers were aligned to various FCoV and FIPV sequences to assure the coverage of circulating variants (Figure S7) and were used for further validation steps.

### 2.2. Analytical Sensitivity and Specifity

**Figure 1:**
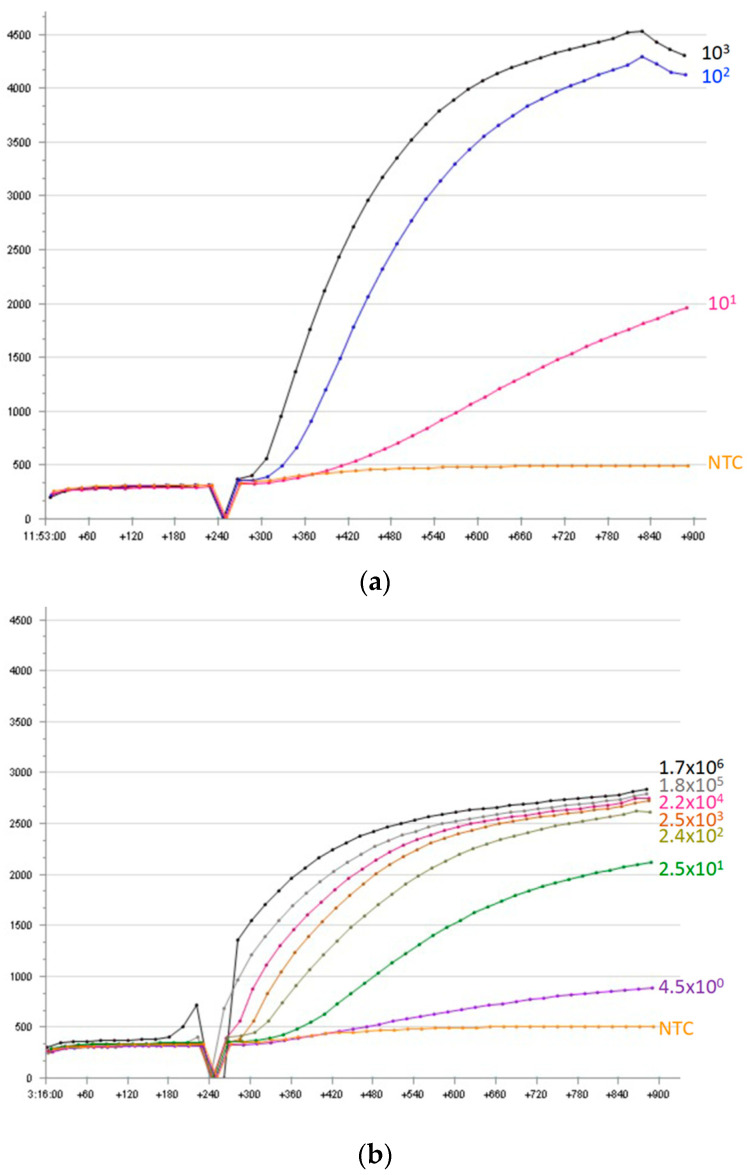
The amplification curves of RT-RPA run with Forward Primer 1 (FP1) and Reverse Primer 3 (RP3) using (**a**) serial dilution of molecular standard from 10^3^ to 10^1^ copies/μL and (**b**) serial dilution of extracted RNA of feline coronavirus supernatant from cell culture (1.7 × 10^6^ to 4.5 × 10^0^ copies/μL) together with negative template control (NTC). The drop in the fluorescence signal after three minutes was due to the mixing step, which is necessary to produce a homogeneous RPA reaction. For each run, 5 μL was used, which lead to a limit of detection of 22.5 × 10^0^ copies/reaction of the viral RNA.

**Figure 2:**
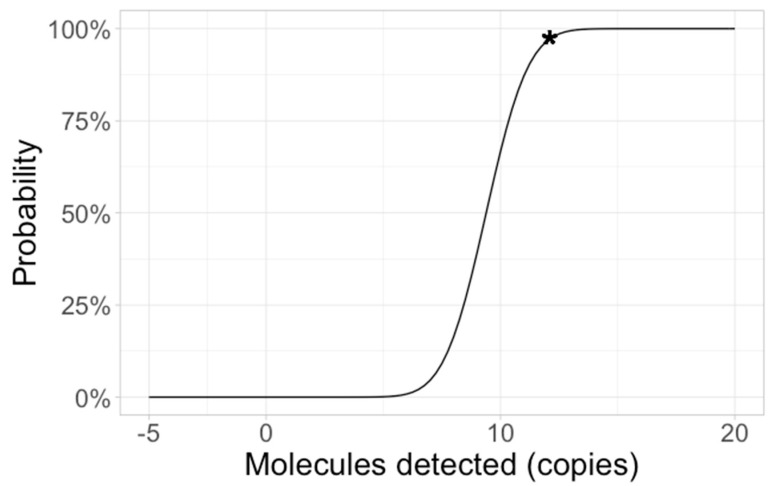
A probit analysis based on the results of nine RT-RPA runs with molecular standard 5 × 10^3^ to 10^0^ RNA copies/reaction. The limit of detection is displayed per microliter (11.7 RNA molecules), which is 58.5 copies/reaction (depicted as asterisk).

The RT-RPA assay’s limit of detection was determined using various concentrations of an in vitro-transcribed RNA molecular standard (5 × 10^3^ to 10^0^ copies/reaction) (Figure 1a). Out of nine RT-RPA runs, 5 × 10^3^ and 5 × 10^2^ copies/reaction were detected 9/9 times; 50 copies/reaction, 6/9, while 5 copies/μL was not detected. A probit analysis based on these results revealed a limit of detection of 58.5 RNA copies/reaction (95% CI) (Figure 2).

Serial dilutions of FCoV RNA extracted from tissue culture supernatant were tested in quantitative RT-PCR. A range of 8.5 × 10^6^ to 22.5 × 10^0^ copies/reaction of RNA was revealed. The FCoV RT-RPA assay amplified down to 22.5 × 10^0^ copies/reaction of the genomic RNA (Figure 1b), which indicates similar analytical sensitivity to the real-time RT-PCR. Additionally, the same serial dilution of the viral RNA was spiked with nucleic acid extracted from a fecal sample that was tested negative using real-time PCR. As in Figure S3, this analytical sensitivity was 50 viral RNA copies/reaction in the presence of host background nucleic acids.

Results achieved with the RT-RAA kit which was used to compare the performance of our oligo mixes and the molecular standard with kits from different producers were the same as with the RPA kit from TwistDx (Figure S4).

Both the RT-RPA assay and real-time RT-PCR showed no cross-detection to the RNA/DNA of 16 different viruses but amplified two coronaviruses in addition to the FIPV (canine coronavirus strain 1–71 Riemser Virusbank (RVB), transmissible gastroenteritis virus strain 70 RVB) (Table 1, Figure S5a–d).

### 2.3. Clinical Samples

The clinical performance of the RT-RPA assay was examined using 39 fecal samples (Table S1). The RT-RPA results were compared with those of the real-time RT-PCR. In total, 22 samples tested positive by real-time RT-PCR, while 20 were tested positive in RT-RPA. Both assays had the same clinical specificity (number of negative samples = 17) (Table 2).

### 2.4. Sequencing

The 3′UTR end of three samples, including one false negative and two positive identified samples, were sequenced. The obtained sequences were compared using Geneious Prime to evaluate whether changes in the nucleotides of one sample were the cause of false negative results in the RT-RPA. The alignment of the sequences showed no differences between the three samples in the primer and probe region.

## 3. Discussion

FCoV infection poses a high threat to cats, especially kittens, due to the possible development of FIP. To prevent further spread of the infection, the development of a fast and sensitive surveillance system for FCoV shedding is a necessity. In this study, an isothermal RT-RPA assay for rapid detection of FCoV within 15 min was developed and its diagnostic utility was compared to real-time RT-PCR. A limit of detection of 58.5 copies/reaction and a clinical sensitivity and specificity of 90.9 and 100%, respectively, were achieved. Of all viruses, RNA viruses in particular have a high tendency to mutate [39]. To detect all different strains of FCoV, the target sequence used for a diagnostic assay should be conserved. Therefore, the untranslated region of the accessory 7b gene (function unknown [40]) at the 3’-end of the viral genome was selected as a target for the FCoV RT-RPA, since it is described as highly conserved among FCoVs [28]. Moreover, its good clinical performance was already proven in many studies [16,41,42].

As no strict rules for RPA primer design are prescribed, several primer combinations were tested in the present study. Many recommendations can be followed from the kit’s producer [43] and/or published data [33,44]. Generally, the size of the primers should be between 30 and 36 bases, and multiple Gs in the first five bases of the 5´-end must be avoided. Furthermore, the recommended GC content is between 20–70%. The complexity of primer design is high in the presence of secondary structure in the target sequence. Moreover, the binding of primers can change the folding of the target region and prevent other oligonucleotides from accomplishing the amplification step [33]. Since coronaviruses have a positive-sense RNA genome [1], a reverse transcriptase step is necessary. Therefore, the reverse primer (RP) is needed for both the RT and the amplification steps. As a consequence, a higher concentration of reverse primers would increase the assay’s overall performance [44]. In the present study, different concentrations of the reverse primer were tested (data not shown) and the highest sensitivity was achieved using a double concentration of the RP. The same phenomenon was observed during the design of the SARS-CoV-2 RT-RPA assays [44].

The cross-detection to other pathogens was determined by using 19 different viruses, which either belonged to the family *Coronaviridae* or were common pathogens in cat and dog populations. Both the new assay and the real-time RT-PCR-amplified strains of FCoV, as well as one strain of canine coronavirus (CCoV) and one strain of transmissible gastroenteritis virus (TGEV), are all *alphacoronaviruses*. Antigenic cross-detection is limited to species that belong to the same genus [1]. Sequence analysis of the 3′ UTR of the accessory 7 gene revealed high similarities between FCoV, CCoV and TGEV [45,46,47]. The most important question is the relevance of such a finding to the clinical field observations. Even though the natural infection of cats with CCoV and TGEV has not been officially reported, cats can be experimentally infected with these viruses and seroconvert after infection [48]. In this regard, positive results of both RT-RPA and real-time RT-PCR should be confirmed by sequencing, especially if cats are living in close contact with other animals, as coronaviruses are known to have crossed species barriers [49].

Of all fecal samples detected positive in real-time RT-PCR (n = 22), twenty samples with Ct values between 15 and 36 in real-time RT-PCR were correctly identified as positive in the RT-RPA assay. The other two positive samples in real-time RT-PCR were false negative in the RT-RPA assay. Generally, false negative results can be due to low viral load, as well as mutations at the targeted sequence or inhibitory effects. In this case, the latter is unlikely to be the reason, as RPA is known for its robustness to inhibitory effects [50]. Low viral load could be the explanation for one of the false negative results, as this sample had a Ct value higher than 35 (Table S1). For the other false negative sample, low viral load is unlikely to be the cause, since the sample had a Ct of 27 and other positive samples with a Ct value of 30 and more were identified correctly in FCoV-RT-RPA. Even though the target sequence is highly conserved, there is a possibility of mutations. To exclude sequence variation as a cause, the corresponding sample was sequenced and then aligned to the sequence of two correctly identified samples using Geneious Prime. No differences in nucleotides were observed. Therefore, the reason for the false negative result remains unclear.

Another very popular isothermal amplification assay is the loop-mediated isothermal amplification (LAMP). LAMP is based on the use of a DNA polymerase with strand displacement activity, making amplification possible at a constant temperature (60–65 °C) for 30–60 min [31]. LAMP requires at least four to six primers which is not easy to design for very limited conserved regions in RNA viruses [51]. Hitherto, three LAMP-based assays for detection of FCoV were published. In one study, screening 71 samples with two commercially available LAMP assays revealed sensitivities of 35.3% and 58.8% [30]. This inferior clinical sensitivity of the RT-LAMP to the RT-PCR is consistent with the results of a previously published study (around 50%) [52]. By contrast, the newly developed RT-RPA assay was fast (main TT value 170 s) and sensitive (90.9%), relying on one primer pair.

Immunochromatography tests for the detection of FCoV antigen are commercially available from various companies. The sensitivity and specificity in comparison to RT-PCR is around 95% [53,54]. Unfortunately, the claimed diagnostic sensitivities were not evaluated by an independent research group.

The standard molecular diagnostic methods for RNA virus detection remains real-time RT-PCR. This method, however, is time-consuming and requires a well-equipped laboratory, rendering it impractical as a routine point-of-need test. To overcome these drawbacks, rapid PCR systems have been developed recently [55,56,57]. The high speed of the PCR was achieved by changing the cycling protocol [57]. One research group optimized the heat transfer to achieve fast thermal cycling with the use of a sample holder that quickly dissipated excess heat due to its high thermal conductivity. The significant reduction of the transition times (68% compared to commercial real-time PCR) leads to an increased speed of amplification [58]. A different approach to speed up PCR is the use of induction heating [59]. All these techniques are still in the early stages of development and no protocols for the detection of FCoV are available yet. In terms of applicability for point-of-need testing, RPA shows clear advantages. The detection device is easy to transport and operate via a solar battery. Results can be achieved in less than 15 min. Additionally, RPA reagents are available in a dry pellet form. These advantages led to the implementation of RPA in a mobile suitcase lab to make diagnosis possible in low-resourced settings for several viruses [33,38,44,60].

One RPA assay for detection of FCoV was developed based on the membrane gene as the target region [61], which was reported highly sensitive and specific. Unfortunately, it was not possible to reproduce these results in our laboratory (Figure S6), which might be due to a difference in the kits’ sources. To make sure that the current FCoV-RT-RPA was compatible with kits from other producers, RT-RAA was performed following manufacturer instructions using our molecular RNA standard and oligo mixes. Exactly the same analytical sensitivity was achieved. That indicates the robustness of our assay. A limitation of the study is that all samples were extracted by silica-based RNA extraction kits before being tested in both real-time RT-PCR and RT-RPA. This step is time-consuming. The inclusion of a simple preparation step will be necessary for field application. This seems very realistic, as the RPA can tolerate inhibitors since crude samples have been used directly in previous studies [62,63]. Furthermore, it is important to emphasize that the designed assay is a good method for screening cat populations for FCoV infections, but is not designed to confirm a FIPV infection.

To conclude, the FCoV-RT-RPA was proven a rapid and sensitive assay for the detection of FCoV in the extracted samples. The deployment of rapid point-of-need tests and following measures would lead to a significant reduction of FCoV in cat populations. The easy handling of the RT-RPA assay makes repeated testing possible for identifying intermittent shedders.

## 4. Materials and Methods

### 4.1. Clinical Samples and Ethical Statement

In total, 39 archived samples were used. Three samples were used from a pool of archived samples from cats with an unknown infection status collected during a surveillance study in Leipzig approved by the Landesdirektion Sachsen: A 19/17. A total of 36 fecal samples were collected during routine diagnosis. The owners had approved the use of the leftovers from the samples for research purposes. To test for cross-detection, inactivated viruses which were not available in our laboratory were provided by Friedrich Loeffler Institute, Robert Koch Institute and Charité Berlin, Germany. Viral RNA from all samples was extracted using the viral RNA Mini Kit (QIAGEN, Hilden, Germany) according to the instructions of the manufacturer.

### 4.2. Molecular RNA Standard

RNA standard (3′ UTR of the 7b gene of FCoV, nucleotides 28,584 to 29,096 of the GenBank accession number DQ010921) was used to determine the assay’s analytical sensitivity. First, a DNA strand was produced by Thermo Fisher Scientific GENEART (Regensburg, Germany) with the T7 promotor attached at the 5′-end (5′–TAATACGACTCACTATAG–3′). The DNA was transcribed into RNA using HiScribe T7 Quick High Yield RNA Synthesis Kit (New England Biolabs GmbH, Frankfurt, Germany) following the manufacturer’s instructions. DNase treatment was applied to remove the background DNA using DNase I (2000 U/mL) (New England Biolabs GmbH, Frankfurt, Germany). RNA quantification was performed by Qubit RNA BR Assay Kit from Thermo Fisher Scientific (Regensburg, Germany). Ten-fold serial dilutions ranging from 10^7^–10^0^ RNA molecules/μL were prepared.

### 4.3. Real-Time RT-PCR

The real-time RT-PCR assay was performed using a published protocol [27] on the Stratagene Mx3005p QPCR system (Agilent Technologies, Santa Clara, CA, United States) using the QuantiTect Probe RT-PCR Kit (QIAGEN, Hilden, Germany). Briefly, the reaction mix contained 10 μL of the 2x QuantiTect Probe RT-PCR Master Mix, 7.5 μL of PCR clean water, 0.2 μL of the QuantiTect RT Mix, 0.5 μL of forward primer FCoV1128f-5′-GATTTGATTTGGCAATGCTAGATTT-3′, 0.5 μL of reverse primer 5′-FCoV1229r 5′-AACAATCACTAGATCCAGACGTTAGCT-3′ and 0.3 μL of the probe 5′-6FAM-TCCgCTATgACgAgCCAACAATggATMR-3′ (each 10 μM) [27]. Then 1 μL of the template was added. The thermal profile was applied as follows: 50 °C for 30 min, 95 °C for 15 min, then 40 cycles of 94 °C/15 s and 60 °C/45 s.

### 4.4. Real-Time RT-RPA

Three forward and three reverse primers as well as Exo-probe (Table 3) were produced by TIB Molbiol (Berlin, Germany) and Biomers (Ulm, Germany). Various oligonucleotide combinations were tested to select the most sensitive RT-RPA assay. The optimal RT-RPA reaction mix was conducted using TwistAmp Exo kit (TwistDx Ltd., Cambridge, UK) and lyophilized Reverse Transcriptase RevertAid from Thermo Fisher Scientific (Regensburg, Germany) as follows: 8.2 μL of Reverse Transcriptase (500 U per reaction), 29.5 μL of rehydration buffer, 2.5 μL of magnesium acetate, 2.1 μL of 10 μM forward primer, 2.1 μL of 20 μM reverse primer, 0.6 μL of 10 μM probe and 5 μL of the RNA template were added into the lid of the tube containing freeze-dried reaction pellets. In each RT-RPA run, one tube with molecular biology grade water was used as a negative control. The tube was closed, spun, mixed, and spun again (SMS). Thereafter, the tube was incubated into the T8-ISO instrument (Axxin, Fairfield, Australia) at 42 °C for 15 min. After 230 s, a mixing step by vortexing was performed. The FAM fluorescence signal intensities were measured every 20 s. The run with the different kit was performed using our molecule standard and oligo mixes with an RT-RAA nucleic acid amplification kit (Fluorescent Method, Jiangsu Qitian Gene Technology Co., Ningbo, China) according to manufacturer instructions.

### 4.5. Analytical Sensitivity and Specificity

The analytical sensitivity of the RT-RPA assay was determined using serial dilution of the molecular RNA standard (10^3^–10^0^ RNA copies per μL) as well as various concentrations of viral whole-genome extracted from cell culture supernatant (1.7 × 10^6^ to 4.5 ×10^0^ RNA copies per μL). Per reaction, 5 μL of the RNA was used. For determining the analytical sensitivity, the viral RNA dilution series was tested with and without host background nucleic acid extracted from real-time RT-PCR FCoV negative samples (nanodrop value: 26 ng/μL for DNA and 21.1 ng/μL for RNA). Using RStudio version 1.3.1093 (RStudio, Boston, MA, United States) [64], a probit regression was performed and the limit of detection was calculated. The illustration was created using the ggplot2 package (v3.3.3; [65]). To determine the cross-detection of the RT-RPA assay, the RNA/DNA of 19 viruses from cell culture were tested (Table 1).

### 4.6. RT-PCR and Sequencing

The sequencing of the 7b gene of three samples was conducted based on a previous publication of Lin et al. [66] RT-PCR was performed using OneStep RT-PCR Kit from (QIAGEN, Hilden, Germany). The reaction mix contained 5 μL of the 5xOneStep RT-PCR Buffer, 10.5 μL of PCR clean water, 1 μL of the dNTP mix, 1.25 μL each of the 10 μM forward (7a–F1: 5′-CTGCGAGTGATCTTTCTAG-3′) and reverse primer (P211: 5′-CACTAGATCCAGACGTTAGCTC-3′) and 1 μL of the OneStep RT-PCR Enzyme Mix. Thereafter, 5 μL of the extracted sample was added. The following thermal profile was used for RT-PCR: 50 °C for 30 min, 95 °C for 15 min, then 35 cycles of 94 °C/30 s, 56 °C/30 s and 72 °C/1 min.

For electrophoresis, 8 μL of each sample was analyzed using a 1.5% agarose gel. The amplicon was purified (NucleoSpin Gel and PCR Clean-up, Mini kit for gel extraction and PCR clean up, MACHEREY-NAGEL, Dueren, Germany) and then sequenced by Eurofins Genomics (Munich, Germany). Geneious prime (2 February 2020, Auckland, New Zealand) was applied for data analysis.

## Figures and Tables

**Table 1:** List of viruses used for determining the cross-detection of the RT-RPA assay. The results are shown in the cycle threshold (Ct) of real-time RT-PCR and time threshold (TT, s.) of RT-RPA. RVB is Riemser Virusbank.

Virus	Real-Time RT-PCR (Ct)	RT-RPA (TT)
Feline infectious peritonitis virus ^++++^	13.42	120
Canine coronavirus strain 1-71 RVB ^+^	15.97	160
Transmissible gastroenteritis virus strain 70 RVB ^+^	18.38	180
Transmissible gastroenteritis virus strain 545 RVB ^+^	No Ct	Neg
Feline calicivirus ^++++^	No Ct	Neg
Feline herpesvirus ^++++^	No Ct	Neg
Feline parvovirus ^++++^	No Ct	Neg
Canine parvovirus ^++++^	No Ct	Neg
Canine herpesvirus ^++++^	No Ct	Neg
Canine minute virus ^++++^	No Ct	Neg
Canine adenovirus ^++++^	No Ct	Neg
Canine distemper virus ^++++^	No Ct	Neg
Bovine coronavirus V321.2 ^+^	No Ct	Neg
Severe acute respiratory syndrome coronavirus ^+++^	No Ct	Neg
Severe acute respiratory syndrome coronavirus 2 ^+++^	No Ct	Neg
Human coronavirus 229E ^++^	No Ct	Neg
Human coronavirus NL63 ^++^	No Ct	Neg
Human coronavirus OC43 ^++^	No Ct	Neg
Middle East respiratory syndrome coronavirus ^++^	No Ct	Neg

^+^ = provided by Friedrich Loeffler Institute (Greifswald, Germany). ^++^ = provided by Robert Koch Institute (Berlin, Germany). ^+++^ = provided by Charité (Berlin, Germany). ^++++^ = Institute of Animal Hygiene and Veterinary Public Health (Leipzig, Germany).

**Table 2:** Clinical sensitivity and specificity of RT-RPA and real-time RT-PCR. n = number of tested samples.

	RT-RPA	Real-Time RT-PCR
Sensitivity (n = 22)	90.9%	100%
Specificity (n = 17)	100%	100%

**Table 3:** List of RT-RPA oligonucleotides. BHQ = Black Hole Quencher, FAM = fluorescein amidite.

Names	Sequences (5′-3′)
Forward primer 1 (FP1)	TCATCGCGCTGCCTACTCTTGTACAGAATGGTAAG
Forward primer 2 (FP2)	CCGATGTCTAAAACTTGTCTTTCCGAGGAATTAC
Forward primer 3 (FP3)	ACTTGAAGCAATTCAGAAGCAAGAAGGTCTTCGAC
Reverse primer 1 (RP1)	AATCTAGCATTGCCAAATCAAATCTAAACTTCCTA
Reverse primer 2 (RP 2)	GTCATAGCGGATCTTTAAACTTCTCTAAATTACTA
Reverse primer 3 (RP 3)	ACTAGATCCAGACGTTAGCTCTTCCATTGTTGGCTC
ExoProbe (P)	ATCTAAACTTCCTAA (BHQ1-dT, Tetrahydrofuran and FAM-dT) GCAATAGGGTTGCTTGTACCTCCTATTACACG--Phosphate

## Data Availability

All data produced in the study is mentioned in the manuscript or Supplementary Materials.

## Supplementary Materials

The following are available online at https://www.mdpi.com/article/10.3390/pathogens10101237/s1, Figure S1: Amplifications curves of RT-RPA testing different primer combinations to achieve the highest sensitivity of the RT-RPA assay. Figure S2: Amplification curves of the different primer combinations using molecular water as a template for control of unspecific fluorescence signal. Figure S3: Amplification curves of RT-RPA using an extracted faecal sample spiked with serial dilution of extracted RNA of feline coronavirus supernatant from cell culture. Figure S4: Amplifications curves of RT-RAA runs using primer FP1 and RP3 designed in this study with the molecular standard dilution range (106 to 10 copies/μL). Figure S5: Amplifications curves of RT-RPA runs with DNA/RNA of 19 viruses extracted from cell culture that were tested in order to determine the cross-detection of the assay. Figure S6: Amplifications curves of RPA runs using primer and probes published in “Development of a recombinase polymerase amplification fluorescence assay to detect feline coronavirus”. Figure S7: Alignment of FP1, RP3 and ExoProbe with the genome sequences of different strains of (a) FCoVs and (b) FIPVs. Table S1: Summary of all samples tested. Results are shown in Cycle Threshold (Ct) for real-time RT-PCR and Time Threshold (TT, s) for RT-RPA.
