# Supplementary material for: Molecular Detection of Feline Coronavirus Based on Recombinase Polymerase Amplification Assay
Source: Pathogens. 2021 Sep 25;10(10):1237. doi: 10.3390/pathogens10101237 (PMC8538120; doi:10.3390/pathogens10101237)
Supplement: Supplementary file 1 [file pathogens-10-01237-s001.zip › pathogens-1350500-supplementary.pdf]

pathogens

Supplementary File

Molecular Detection of Feline Coronavirus Based on Recombinase Polymerase Amplification Assay

Rea Maja Kobialka [1], Arianna Ceruti [1], Michelle Bergmann [2], Katrin Hartmann [2], Uwe Truyen [1] and Ahmed Abd El Wahed [1,*]

[1] Institute of Animal Hygiene and Veterinary Public Health, Leipzig University, 04103 Leipzig, Germany; rea_maja.kobialka@uni-leipzig.de (R.M.K.); arianna.ceruti@uni-leipzig.de (A.C.); truyen@vetmed.uni-leipzig.de (U.T.)
[2] Clinic of Small Animal Medicine, LMU, 80539 Munich, Germany; N.Bergmann@medizinische-kleintierklinik.de (M.B.); hartmann@medizinische-kleintierklinik.de (K.H.)
* Correspondence: ahmed.abd_el_wahed@uni-leipzig.de; Tel.: +49-341-97-38-153

Table S1. Summary of all samples tested. Results are shown in Cycle Threshold (Ct) for real-time RT-PCR and Time Threshold (TT,sec.) for RT-RPA.

cat	*sample*	*real-time RT-PCR (Ct)*	*RT-RPA (TT)*
1	faecal	20.09	340
2	faecal	24.67	320
3	faecal	28.95	280
4	faecal	28.00	280
5	faecal	24.25	280
6	faecal	34.14	280
7	faecal	30.75	280
8	faecal	23.01	280
9	faecal	29.24	280
10	faecal	27.56	280
11	faecal	23.63	280
12	faecal	28.85	280
13	faecal	33.51	200
14	faecal	24.46	180
15	faecal	19.58	180
16	faecal	19.85	160
17	faecal	19.29	160
18	faecal	19.90	140
19	faecal	19.65	140
20	faecal	16.93	120
21	faecal	35.02	-
22	faecal	27.27	-
23	faecal	No Ct	-
24	faecal	No Ct	-
25	faecal	No Ct	-
26	faecal	No Ct	-
27	faecal	No Ct	-
28	faecal	No Ct	-
29	faecal	No Ct	-
30	faecal	No Ct	-
31	faecal	No Ct	-
32	faecal	No Ct	-
33	faecal	No Ct	-
34	faecal	No Ct	-
35	faecal	No Ct	-
36	faecal	No Ct	-
37	faecal	No Ct	-
38	faecal	No Ct	-
39	faecal	No Ct	-

(a)

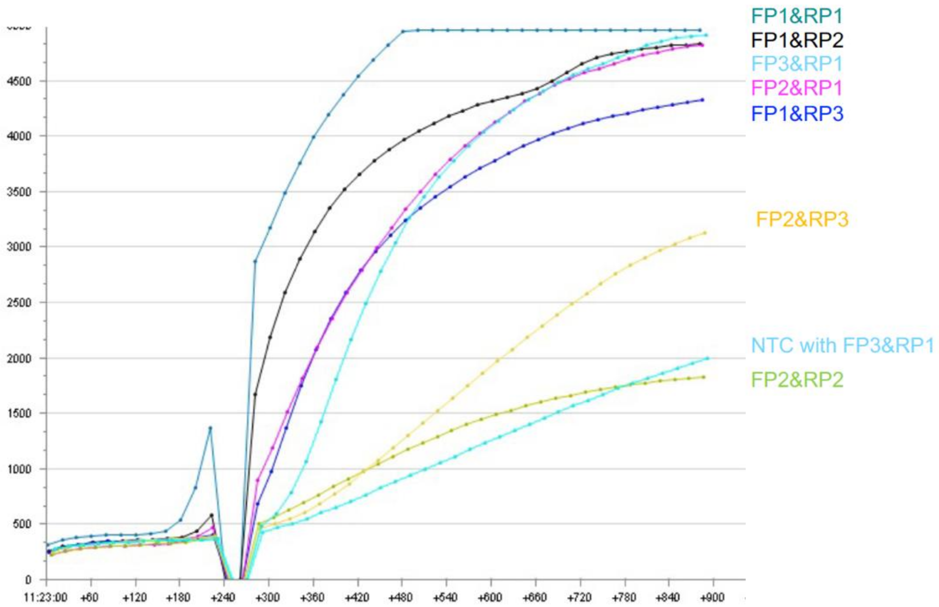

(b)

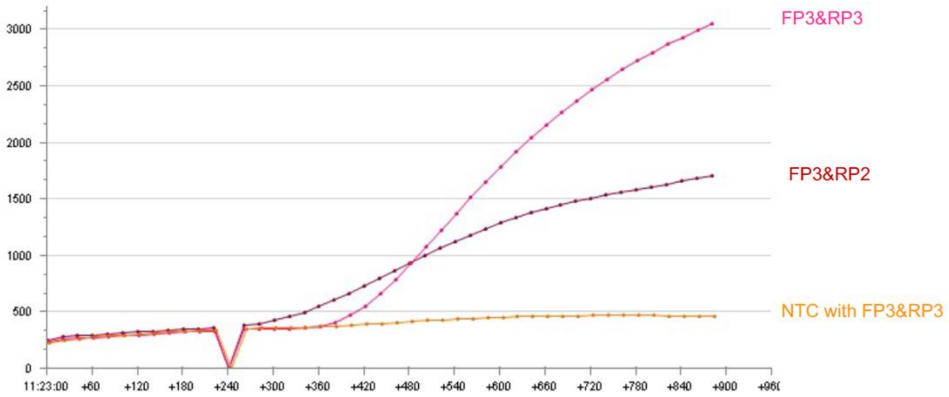

(c)

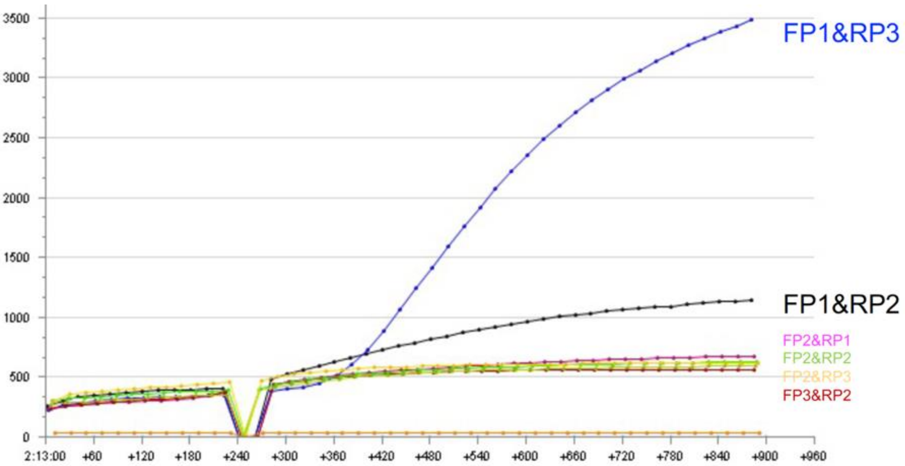

Figure S1. Amplifications curves of RT-RPA testing different primer combinations to achieve the highest sensitivity of the RT-RPA assay. (**a&b**) Molecular standard 10^5 copies/ µL and (**c**) molecular standard 10^2 copies/ µL were used as template.

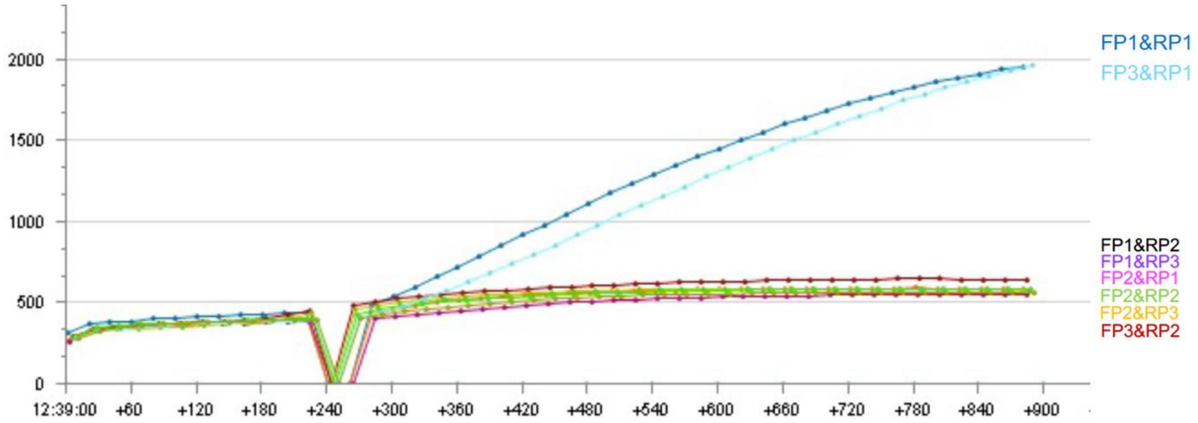

Figure S2. Amplification curves of the different primer combinations using molecular water as a template for control of unspecific fluorescence signal.

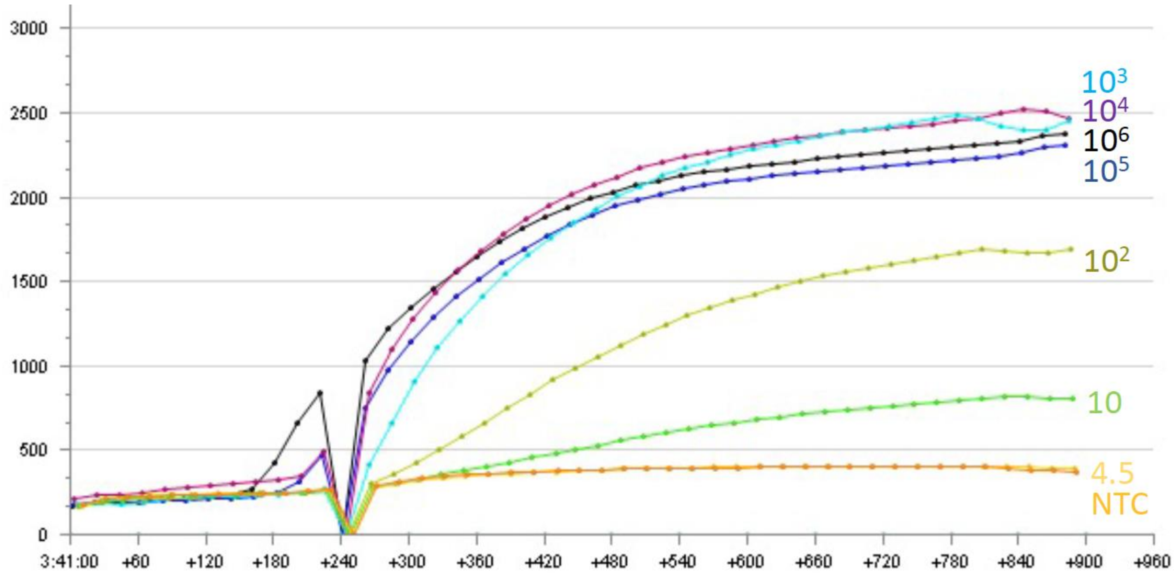

Figure S3. Amplification curves of RT-RPA using an extracted faecal sample spiked with serial dilution of extracted RNA of feline coronavirus supernatant from cell culture. Negative template control (NTC) is molecular biology water.

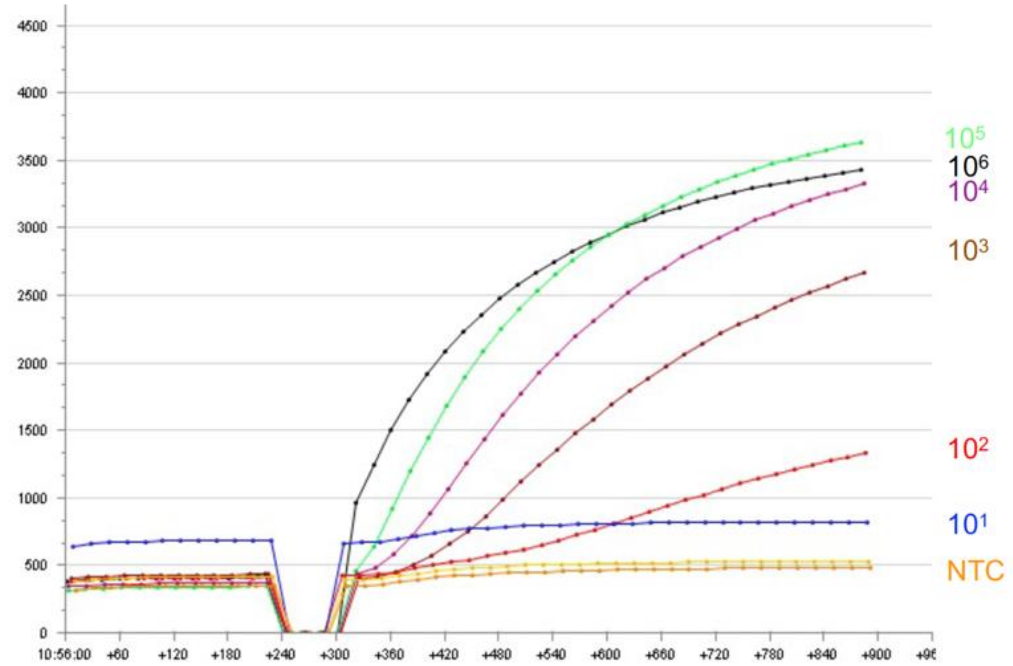

Figure S4. Amplifications curves of RT-RAA runs using primer FP1 and RP3 designed in this study with the molecular standard dilution range (10^6 to 10 copies/ μL).

(a)

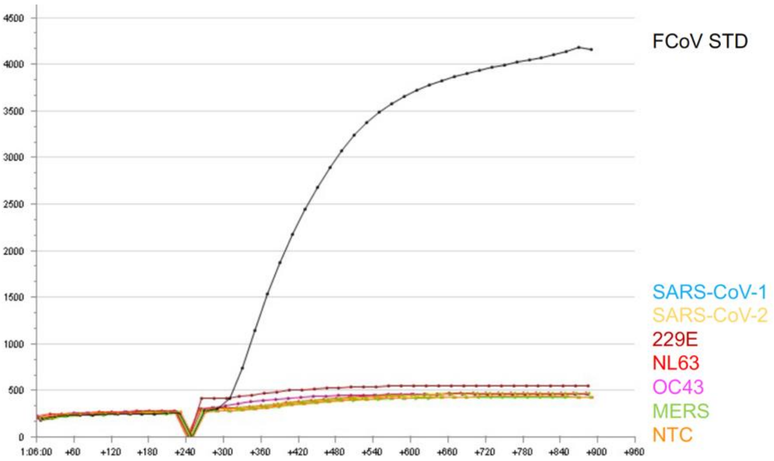
FCoV STD
SARS-CoV-1
SARS-CoV-2
229E
NL63
OC43
MERS
NTC

(b)

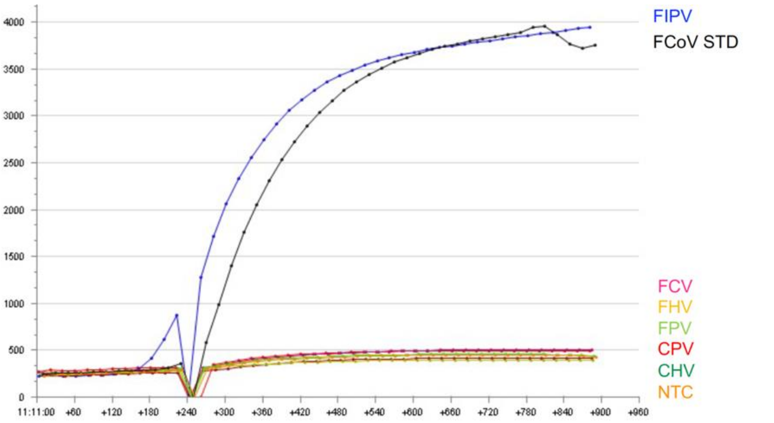
FIPV
FCoV STD
FCV
FHV
FPV
CPV
CHV
NTC

(c)

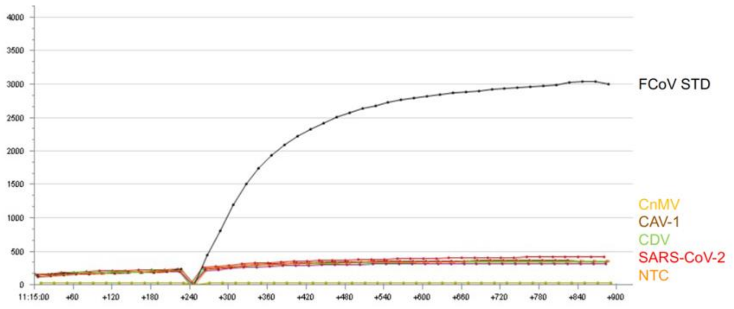
FCoV STD
CnMV
CAV-1
CDV
SARS-CoV-2
NTC

(d)

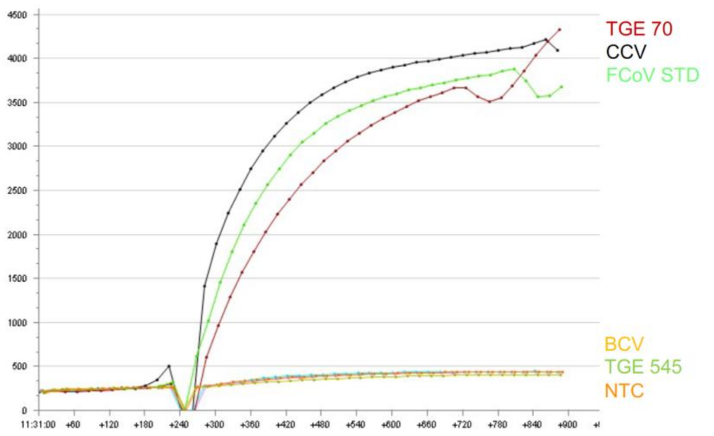

Figure S5. Amplifications curves of RT-RPA runs with DNA/RNA of 19 viruses extracted from cell culture that were tested in order to determine the cross-detection of the assay.

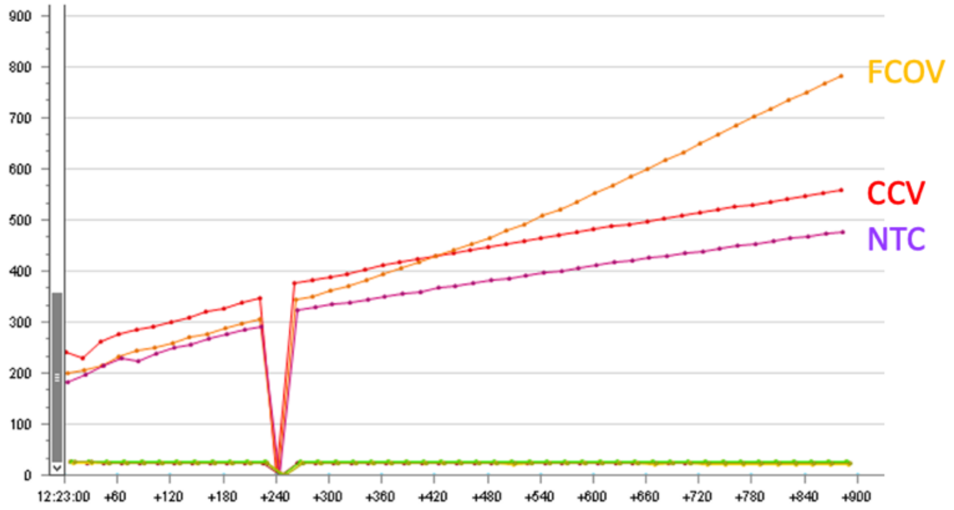

Figure S6. Amplifications curves of RPA runs using primer and probes published in "Development of a recombinase polymerase amplification fluorescence assay to detect feline coronavirus" Hu et al. with extracted RNA of feline coronavirus and canine coronavirus supernatant from cell culture.

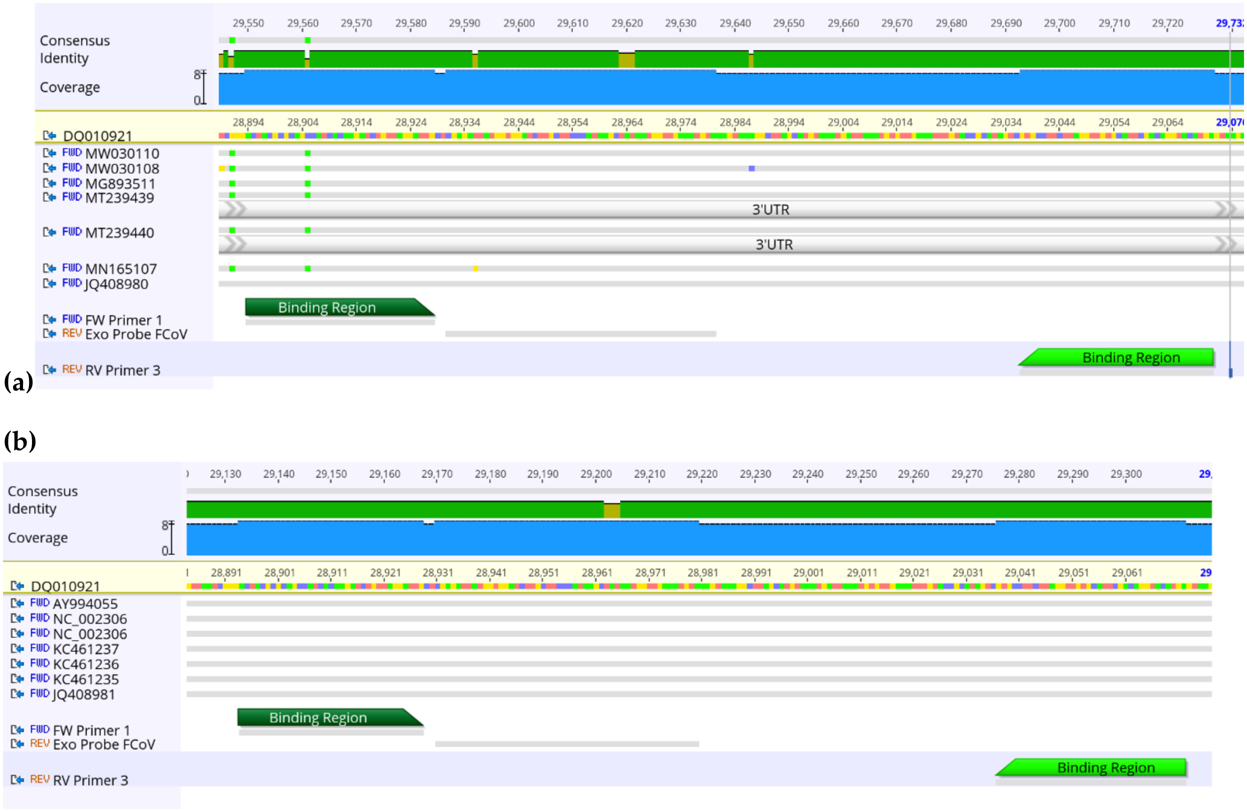

Figure S7. Alignment of FP1, RP3 and ExoProbe with the genome sequences of different strains of (a) FCoVs and (b) FIPVs. The alignment was created with Geneious Prime. The ID is the GenBank accession number.
